# O4.6. GENOME-WIDE ASSOCIATION STUDY, HERITABILITY ESTIMATION AND POLYGENIC RISK ANALYSIS OF SUSCEPTIBILITY TO INFECTIONS IN 65,534 INDIVIDUALS WITH SEVERE MENTAL DISORDERS AND POPULATION CONTROLS

**DOI:** 10.1093/schbul/sby015.212

**Published:** 2018-04-01

**Authors:** Michael Benros, Ron Nudel, Yunpeng Wang, Vivek Appadurai, Andrew Schork, Esben Agerbo, Thomas Werge, Merete Nordentoft, Preben Mortensen, Alfonso Buil, Wesley Thompson

**Affiliations:** 1 Mental Health Centre Copenhagen; 2 Institute of Biological Psychiatry, Mental Health Center St. Hans, Mental Health Services Copenhagen; 3 National Centre for Register-based Research, Aarhus University; 4 Mental Health Centre Copenhagen, Copenhagen University Hospital

## Abstract

**Background:**

Infections are one of the major disease burdens internationally; however, the genetic architecture of infections is largely unknown. The human leukocyte antigen (HLA) loci have been implicated in susceptibility for infections; however, to date, largescale HLA type and genome wide association studies (GWAS) of infections have been lacking. We aim to investigate the genetic architecture of infections with GWAS of single-nucleotide polymorphisms (SNPs) and HLA types, including associations with mental disorders.

**Methods:**

We conducted case-cohort association analysis using both SNP’s and HLA types from a Danish population-based sample born after 1981 comprising of 65.534 unrelated Danish individuals. All individuals were linked utilizing nationwide population-based registers with virtually complete registration of all hospital contacts for infections from birth, where 28.472 (43%) individuals had ≥1 infection requiring hospitalization. Among the 45.889 cases with mental disorders, a total of 21.728 (47%) cases had hospitalizations for infections, whereas among the 19.645 individuals with no severe mental disorders, a total of 6.744 (34%) cases had hospitalizations for infections. All analyses were adjusted for age, sex, and principal components.

**Results:**

We will present GWAS findings of the overall susceptibility for acquiring infections among individuals with severe mental disorders exploring differences to population controls. Furthermore, we will present SNP heritability of acquiring infections among individuals with severe mental disorders exploring differences to population controls. Moreover, we will present findings from association analysis of HLA types investigating the role of HLA alleles in susceptibility to infections and mental disorders. Lastly, we will present results on possible gene-infection interaction regarding the risk of mental illness.

**Discussion:**

Our findings will illuminate the genetic architecture of acquiring infections, and the genetic associations with mental disorders exploring the possible genetic component of the known association between infections and severe mental disorders, such as schizophrenia. Furthermore, we will for the first time in a large population based study explore the associations with HLA alleles to infections and severe mental disorders.

